# Chemotherapy-induced Takotsubo cardiomyopathy, a case report and review of the literature

**DOI:** 10.1186/s12885-017-3384-4

**Published:** 2017-06-02

**Authors:** Matteo Coen, Fabio Rigamonti, Arnaud Roth, Thibaud Koessler

**Affiliations:** 1 Department of Internal Medicine, Geneva University Hospitals, Geneva, Switzerland; 2 Department of Internal Medical Specialties, Division of Cardiology, Geneva University Hospitals, Geneva, Switzerland; 3 Department of Internal Medical Specialties, Division of Oncology, Geneva University Hospitals, rue Gabrielle Perret-Gentil 4, 1211 Geneva 14, Switzerland

**Keywords:** Takotsubo cardiomyopathy, Chemotherapy, Intra-arterial chemotherapy, Rechallenge, Case report

## Abstract

**Background:**

Several chemotherapy molecules, monoclonal antibodies and tyrosine kinase inhibitors, have been linked to Takotsubo cardiomyopathy (TC).

**Case presentation:**

In this article, we describe the case of a 45-year-old woman who developed TC after receiving an intra-arterial and intra-venous polychemotherapy for locally advanced epidermoid carcinoma of the anal canal. This is the first described case of TC associated with intra-arterial chemotherapy.

**Conclusions:**

A review of the literature points to 5-fluorouracil as the most common molecule associated with TC and highlights the potential risk associated with rechallenging patient with the same drug.

## Background

For oncologists challenging questions remain after TC diagnosis: is the chemotherapy responsible for the TC? Most importantly, how should we treat the patient next? This last question is especially difficult in a curative setting like our case.

We describe a case of nonfatal chemotherapy induced TC in a woman diagnosed with locally advanced epidermoid carcinoma of the anal canal. The TC developed after intra-arterial chemotherapy with cisplatin, 5-fluorouracil, methotrexate, mitomycin, and intra-venous bleomycin. Intra-arterial chemotherapy is very effective at closing anal cancer-related fistulas before the initiation of standard chemoradiation; this strategy has the advantage of increasing the concentration of chemotherapeutics locally. The intra-arterial chemotherapy regimen consisted of cisplatin (8.5 mg/m^2^), 5-fluorouracil (275 mg/m^2^), methotrexate (27.5 mg/m^2^), mitomycin (1.2 mg/m^2^), four intra-arterial infusions per day for two days, and a single intravenous dose of bleomycin (10 mg) on the first day [1].

This is the first report of TC occurring after intra-arterial infusion chemotherapy. TC is a rare and unpredictable event in oncology which needs to be recognized by oncologists as fatal cases of TC are reported when patients are rechallenged with the same molecule.

## Case presentation

**Fig. 1:**
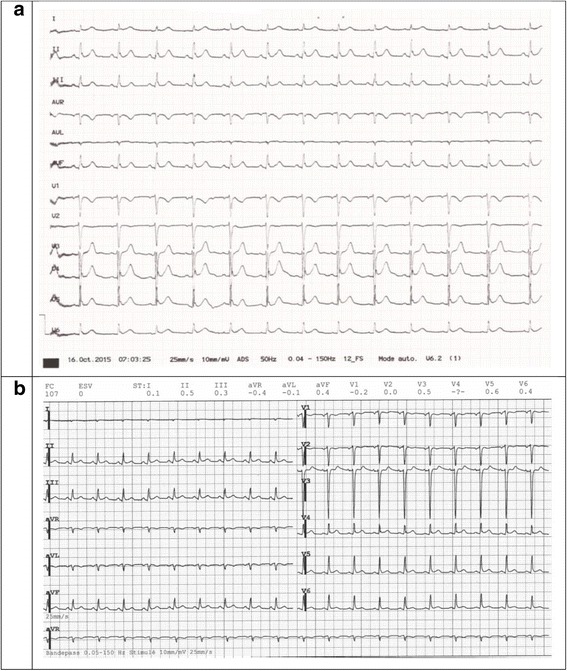
ECG during (**a**) and after haemodynamic instability (**b**). ECG interpretation (during the hemodynamic instability, **a**): Sinus rhythm with 78 bpm, PR of 200 ms, QRS of 100 ms and QT of 360 ms; interventricular conduction delay and signs of early repolarization in V4-V6 as well as II, III et aVF; presence of a negative T wave in V1 concordant with the QRS axe; precordial transition in V4. The ECG after haemodynamic instability (**b**) was performed 24 h after the first one (**a**)

A 45-year-old woman was admitted to the Oncology Unit to receive an induction intra-arterial chemotherapy for a locally advanced epidermoid carcinoma of the anal canal (T4NxMx), before the initiation of standard radio-chemotherapy with 5-fluorouracil and mitomycin C [1, 2]. The day after her chemotherapy, she developed nausea and vomiting accompanied by a poorly defined thoracic pain symptoms. Vital signs and physical examination were unremarkable. Symptoms were controlled by ondansetron. Later on the same day, she developed two episodes of oppressive retrosternal pain radiating to the right shoulder. At this time, the ECG (Fig. 1a) demonstrated 1.5 to 2 mm ST-segment elevation in leads V4 and V5; cardiac troponin T levels were elevated to 349 ng/l (normal values <14 ng/l). Shortly after, the patient developed hypotension (systolic blood pressure: 70 mmHg, Mean Arterial Pressure: 57 mmHg) and lost consciousness. Hypotension was refractory to aggressive fluid resuscitation (cardiogenic shock). The patient was admitted to the Intensive Care Unit (ICU) for aminergic support. Emergency coronary angiography showed no evidence of coronary artery disease or spasm. Transthoracic echocardiogram (TTE) revealed a reduced left ventricular ejection fraction (LVEF) of 30%, with apical and midventricular akinesis and preserved right ventricular function. Within 48 h, evolution was favorable, ECG normalized (Fig. 1b) and the patient was discharged from the ICU to the general ward. Low-dose metoprolol and lisinopril was initiated. The rest of the hospital course was uneventful and the patient was discharged home 13 days after chemotherapy.

TTE, performed 2 weeks after discharge showed normal LV function with LVEF of 60-65%. The diagnosis of TC was retained, according to the Mayo Clinic diagnostic criteria: 1. left ventricular mid segments akinesis with apical ballooning, 2. absence of angiographically evidence coronary disease or plaque rupture, 3. new ECG abnormalities with elevation in cardiac troponins; 4. absence of pheochromocytoma and myocarditis. [3, 4]. The patient was totally asymptomatic and both beta-blockers and ACE inhibitors were stopped. No other trigger except chemotherapy was found. She was not rechallenged with intra-arterial chemotherapy. A month after being discharged, she started chemoradiotherapy. She was given mitomycin only, due to the possible involvement of 5-fluorouracil, and to avoid any risk she was hospitalized to receive her chemotherapy and kept under close cardiac monitoring. The chemo-radiation was uneventful.

Due to the poly-chemotherapy used we are unable to identify for sure the agent responsible. 5-fluorouracil and cisplatin have well-known cardiotoxic effects. Although less common, cardiotoxicity has been reported to occur also with methotrexate, mitomycin and bleomycin [5]. Because 5-fluorouracil is the most commonly incriminated molecule we can only conjecture that it is the culprit in our case. Of note, our patient did not appear to be in psychological distress, making the psychological trigger less likely.

## Discussion & Conclusions

TC (stress cardiomyopathy, apical ballooning syndrome, broken heart syndrome) is a form of reversible cardiomyopathy first described by Sato and colleagues in 1990 [6]. TC mimics myocardial infarction (chest pain, ECG abnormalities, cardiac troponin elevation) and is characterized by an acute and reversible dysfunction of the left ventricle (left ventricular mid segments akinesis with apical ballooning) in the absence of angiographically evidence coronary disease or plaque rupture [7].

TC occurs most commonly in postmenopausal women (5.2/100000 women versus 0.6/100000 men), in a community setting or in a healthcare-related setting [8, 9]. Two percent of patients referred to the hospital for an acute coronary syndrome are diagnosed with TC [10]. Stress, either psychological (“primary form” of TC, up to ≈27% of the cases) or physical (“secondary form” of TC) is a common trigger of TC, however, for many cases no trigger is found. Hypertension, hyperlipidemia, diabetes mellitus, smoking and family history of cardiovascular disease are known TC risk factors, but their implication in the pathogenesis of TC is unclear [11].

TC has a low mortality rate (1-3%) and most have completely recovered in a few weeks; a ≈ 10% recurrence is reported [8, 12]. Notably, a high-risk TC characterized by unfavorable clinical outcomes both in the short and in the long term has recently been described [11].

TC pathogenesis is unclear; different paths have been suggested such as: increased sympathetic activity, coronary spasm, microvascular dysfunction, acute coronary syndrome with reperfusion injury, myocardial microinfarction, impaired myocardial fatty acid metabolism, oestrogen deficiency [13]. The most credited hypothesis is stress-induced release of cathecolamines, resulting in microvascular dysfunction and/or direct myocardial toxicity finally leading to myocardial stunning. Reduced estrogen levels in menopausal women may render the heart more vulnerable to catecholaminergic stress, thus explaining the higher frequency of TC in this population [14].

Several reports have described the occurrence of TC during cancer treatment either by chemotherapy, tyrosine kinase inhibitors or monoclonal antibodies (Table 1). The occurrence of TC during oncologic treatments is commonly attributed to direct cardiotoxicity of the treatment (mostly via free radicals-induced cardiac myocyte damage and death) [15]. Other hypotheses such as a paraneoplastic phenomenon or a cancer-related stress (either psychological or physical, i.e. related to treatment or diagnostic procedures) have been described [16].

**Table 1:** Oncology drugs associated with stress cardiomyopathy

Oncology drug(s)	References
5-Fluorouracil	Cheriparambil KM, et al. Angiology. 2000 [23] Dalzell JR, et al. Anticancer Drugs. 2009 [24] Gianni M, et al. Blood Coagul Fibrinolysis. 2009 [35] Kobayashi N, et al. J Nippon Med Sch. 2009 [21] Stewart T, et al. Intern Med J. 2010 [22] Basselin C, et al. Pharmacotherapy. 2011 [25] Radhakrishnan V, et al. J Pediatr Hematol Oncol. 2011 [19] Grunwald MR, et al. J Clin Oncol. 2012 [26] Ozturk MA, et al. Blood Coagul Fibrinolysis. 2013 [17] Knott K, et al. Int J Cardiol. 2014 [20]
Capecitabine	Qasem, et al. Am J Ther. 2014 [27] Y-Hassan S, et al. Cardiovasc Revasc Med. 2013 [28]
Cytarabine	Madias JE. Oncol Res Treat. 2015 [40] Baumann S, et al. Oncol Res Treat. 2014 [37]
Cisplatin, docetaxel	Toyooka S, et al. Gen Thorac Cardiovasc Surg. 2012 [38]
Cytarabine, daunorubicin	Goel S, et al.World J Clin Cases. 2014 [33]
Paclitaxel, hydroxyurea, 5-Fluorouracil, concurrent radiation	Schweizer MT, et al. J Clin Oncol. 2011 [41]
Axitinib	Ovadia D, et al. J Clin Oncol. 2015 [30]
Sunitinib	Numico G, et al. J Clin Oncol. 2012 [39]
Bevacizumab	Franco TH, et al. Ther Clin Risk Manag 2008 [31]
Rituximab	Smith SA, et al. Heart Fail Clin. 2013 [15] Ng KH, et al. BMJ Case Rep. 2015 [32]
Trastuzumab	Burgy M, et al. Anticancer Res. 2014 [42] Khanji M, et al. Clin Oncol (R Coll Radiol) 2013 [43]
Cetuximab, irinotecan	Kim L, et al. J Invasive Cardiol. 2008 [29]
Combretastatin	Bhakta S, et al. Clin Cardiol. 2009 [18]

Since 2000, 27 cases of cancer therapy-related TC have been reported in the literature, 19 attributed to chemotherapy (Table 1), 6 to monoclonal antibody and 2 to tyrosine kinase inhibitor therapies (Table 1). Amongst these 27 cases, 11 (40.7%) had 5-fluorouracil as part of the regiment, 8 were poly-chemotherapies. 5-fluorouracil is the most common drug associated to TC probably due to the ubiquitous use of this drug in oncology. Ozturk et al., have proposed a possible pathogenic mechanisms of TC due to 5-FU involving the formation of circulating microthrombi due to a 5FU-mediated kallikrein-thrombin generation finally leading to ventricular dysfunction [17].

Of the 27 TC, 14 (52%) were male and 13 (48%) female. The median age was 60.0 years and the mean age 53.6 (standard deviation of ±23.7 years). Cardiovascular risk factors were reported in 11 patients (40.7%): including smoking (3/11; 27.2%), hypertension (5/11; 45.4%), dyslipidemia (3/11; 27.2%) and diabetes (2/11; 18.1%). TC tends to occur early after therapy administration. Of 26 TC cases, 4 (15.4%) occurred during treatment administration, and 14 (61.5%) within the first 6 days (hours: mean 45.3, median 38.0, standard deviation ±40.7) [17–34]. A later occurrence, (between 2 to 6 weeks) has been observed in 5 cases (15.4%) [31, 35–38]*.* One case occurred 4 years after tyrosine kinase inhibitor administration [39].

In 12 cases, it is not known whether rechallenge with the incriminated molecule was attempted. In 15 cases, 12 patients were deliberately changed to another regime, 3 patients were rechallenged with the same regimen. In 2 cases, patients suffered a cardiac arrest during or soon after infusion of 5-fluorouracil and platinum agent; despite prompts cardiopulmonary resuscitation the patient described by Radhakrishnan et al. died [19, 25]. In one case (an 85-year-old woman with diffuse large B cell lymphoma), the rechallenge with R-CHOP under close cardiac surveillance and using incrementally increasing doses of doxorubicin was successful [34].

In conclusion, TC is a rare and unpredictable event among oncologic patients. Nevertheless, patients under significant stress (physical or psychological) like the oncologic once and those with cardiovascular risk factors complaining of cardiac symptoms in particular within the first 6 days of their oncologic treatment should be carefully examined for signs of TC. The most frequent agent linked to TC is 5-fluorouracil. Rechallenging should be avoided.

## Abbreviations

ECG: Electrocardiogram
ICU: Intensive care unit
LV: Left ventricular
LVEF: Left ventricular ejection fraction
R-CHOP: Rituximab, cyclophosphamide, doxorubicin, vincristine, prednisone
TC: Takotsubo cardiomyopathy
TTE: Transthoracic echocardiogram
